# The challenges of oral drug delivery via nanocarriers

**DOI:** 10.1080/10717544.2018.1501119

**Published:** 2018-11-03

**Authors:** Jonas Reinholz, Katharina Landfester, Volker Mailänder

**Affiliations:** a Department of Dermatology, University Medical Center of the Johannes Gutenberg-University Mainz, Mainz, Germany;; b Max Planck Institute for Polymer Research, Mainz, Germany

**Keywords:** Nanocarrier, drug delivery, barrier, transport, biodegradable nanomaterial, transcytosis, exocytosis

## Abstract

The oral application of pharmaceuticals is unarguably the most convenient method of application. Especially for protein- or peptide-based drugs, however, the effectiveness is significantly reduced due to enzymatic digestion in the stomach as well as a poor bioavailability in the small intestine. For these difficult formulations, the encapsulation into nanocarriers would protect the sensitive drug and thus could considerably improve the efficiency of oral drug delivery. In the last years, many candidate biodegradable nanomaterials for such carrier systems have been published. However, before the cargo can be released, the nanocarrier needs to cross multiple barriers of the human body, including a layer of intestinal mucus and epithelial as well as endothelial cells. For overcoming these cellular barriers, transcytosis is favored over a paracellular transport for most nanomaterials as paracellular transport routes lack selectivity of transported molecules once opened up. The exact mechanisms behind the transcellular translocations are up to now still not completely understood. For the vast majority of nanocarriers, the rate of transcellular transport is not sufficient to realize their application in oral drug delivery. Especially trafficking into the endolysosomal pathway often marks a key problem. In this review, we focus on the molecular mechanisms of overcoming cellular barriers, especially transcytosis, and highlight difficulties of oral drug delivery via nanocarriers.

## Why do we need oral drug delivery via nanocarriers?

For the majority of people worldwide, the oral administration of pharmaceuticals is the preferred method of application. Despite the obvious advantage of being pain free, it also offers the feature of being noninvasive and overall convenient to handle. Taking the pharmaceutical market of Germany as an example, in 2017, 120,257 of 163,478 (74%) drugs are available for the peroral application (Abdata.de, 2017). This includes several types of tablets, powders, granulates, drops, and syrups.

However, the oral dosage form also has several drawbacks. Before the orally applied drug is able to reach its target, in most instances it needs to overcome multiple compartments of the human body, which is challenging for a broad spectrum of pharmaceuticals, especially for protein- or peptide-based ones. In general, the first major challenge for the drug after ingestion is surviving the harsh acidic pH value in the stomach. In addition, the proteases pepsin and cathepsin start to digest proteins into peptides. Once the drug surpasses the stomach and enters the small intestine via the duodenum, it faces the major enzymatic digestion machinery of the human body. Oligosaccharides and maltose are degraded into glucose, fructose, galactose, and mannose via sucrase, maltase, and lactase. Lipids are cleaved into glycerol and fatty acids via the pancreatic triacylglycerol lipase and carboxyl ester lipase. Peptides are digested into amino acids via trypsin, chymotrypsin, carboxypeptidase, dipeptidase, and aminopeptidase.

Upon surviving these two major locations of digestion in the human body, the drug needs to be absorbed primarily via enterocytes in the small intestine to reach the bloodstream. Up to now, many pharmaceuticals exhibit a fairly low resorption percentage, resulting in a poor bioavailability (Mei et al., 2013).

Thus, preventing the drug from degradation and enhancing the absorption rate in the small intestine highly improves the impact of orally applied pharmaceuticals. Up to now, many drugs are formulated with an enteric coating. This coating mostly consists of a polymeric layer, which is stable at low pH values and therefore protects the active ingredient from dissolving in the stomach. Upon reaching the rather alkaline pH milieu in the small intestine, the enteric coating breaks down and the drug gets accessible. At this point, it can either have the desired effect directly in the small intestine or get absorbed by enterocytes and transported into the bloodstream, from where it can reach its target cells. Nevertheless, even with such a coating, many pharmaceuticals still exhibit a poor bioavailability via the oral administration route. A prominent example for this problem is the peptide hormone insulin, which is crucial for the glucose absorption from blood. If a patient is suffering from insulin-dependent types of diabetes mellitus, he or she needs to regulate his blood sugar levels via subcutaneous injections of insulin multiple times each day. For millions of people worldwide living with diabetes mellitus (422 million in 2014, World Health Organization, 2016]), an oral dosage form of insulin would significantly increase the quality of life. Up to now, however, there is no orally applied insulin available since the majority of the peptide hormone gets degraded via proteases before it can reach the target cells.

One of the most promising approaches to overcome the aforementioned obstacles for oral drug delivery is the employment of nanomedicines . Nanomedicines can be defined as either nanoscale (<100 nm) imaging agents or therapeutic agents that lead to a systematic enhancement, protection, controlled release, precise targeting, or less cytotoxicity of a drug. During the last years, the range of different nanomedicines has been considerably expanded. In 2016, there were already 51 FDA-approved nanomedicines available and 77 nanomedical products in clinical trials (Bobo et al., 2016). Currently, the most clinically relevant nanomedicines are liposomal, protein-based, polymeric/micelle-based, iron oxide, silica, and gold nanoparticle (NP) formulations (Anselmo & Mitragotri, 2016; Bobo et al., 2016).

For oral drug delivery, especially the encapsulation or complexation of a drug into nanomaterials is relevant. The result, a nanocarrier, is ideally able to: (1) protect the drug from the harsh conditions in stomach and intestine, (2) increase the intestinal absorption into the bloodstream, (3) target specific cells in the human body, and (4) guarantee a controlled release inside the target cells.

In contrast to the fact that most of the drugs worldwide get manufactured for the oral application, nearly all of the FDA-approved nanomedicines are dependent on intravenous injection. Up to date, there is no FDA-approved nanocarrier designed specifically for oral drug delivery. There are, however, several *in vivo* animal studies that show an increased bioavailability of encapsulated or complexed drugs after oral administration. For example, the complexation of daidzein into lipid nanocarriers and subsequent oral administration yielded an increase in bioavailable daidzein in the blood by at least 10-fold over the oral administration of free daidzein in rat studies (Zhang et al., 2011). The encapsulation of insulin into (chitosan-coated) solid lipid NPs resulted in a major boost in relative pharmacological bioavailabilities of 8% (uncoated) and 17% (chitosan-coated) of insulin in rats (Fonte et al., 2011). The oral bioavailability of probucol, a lipophilic drug, could be increased by approximately 10-fold after incorporation into a porous starch based self-assembled nanodelivery system in rat experiments (Zhang et al., 2013).

Nevertheless, the discrepancy between a high number of examined nanocarriers in animal studies and the absence of FDA-approved nanocarriers for oral drug delivery indicates major difficulties in the development of such carrier systems for the human body. This review focuses on these major obstacles for oral drug delivery via nanocarriers, highlighting the importance to cross natural barriers of the human body.

## Natural barriers of the human body and their implications for oral drug delivery via nanocarriers

**Figure 1.**
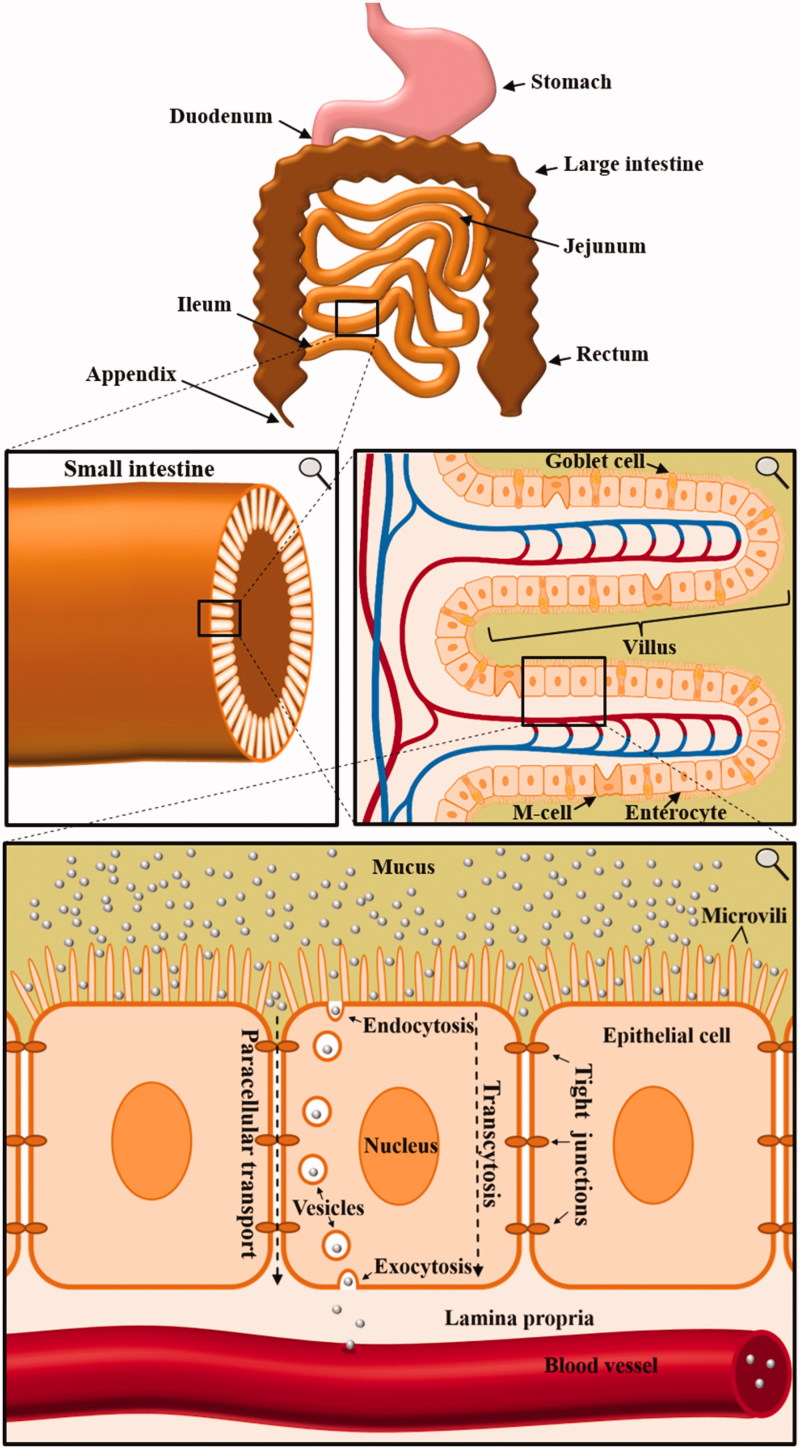
Anatomy of the small intestine and the implications for orally applied nanocarriers. Multiple consecutive close-ups are displayed. In order to enter the bloodstream, orally applied nanocarriers have to cross multiple borders of the human body. Especially the uptake and crossing of enterocytes in the small intestine mark a key challenge in oral drug delivery via nanocarriers.

The major obstacle for oral drug delivery via nanocarriers is the fact that the nanocarrier needs to cross several natural barriers of the human body before the incorporated drug can reach the target cell. Once being ingested, the nanomaterial will protect the drug from the acidic milieu and the proteolytic ‘thunderstorm’ in the stomach. After leaving the stomach, the nanocarrier enters the small intestine and is transported along the duodenum, jejunum, and ileum (Figure 1). Here, either the cargo needs to be released for intestinal absorption or the nanocarrier itself needs to be taken up. Otherwise, the nanocarrier will inevitably be withdrawn from the human body, since the colon does not have the capacity to absorb solid materials. While the small intestine is adsorbing the nutrients, the colon afterward solely reduces the amount of water in the feces. Following this uptake in the small intestine, the nanocarrier needs to traverse the layer of epithelial cells to reach the lamina propria. From there on, the next obstacle is a layer of endothelial cells of the blood vessel, which the nanocarrier needs to transverse in order to reach the lumen of the blood vessel. Once the nanocarrier enters the bloodstream, there are two possibilities. Either the drug can be directly released into the blood stream or further transported to a target cell. However, the second possibility bears two additional barriers – the traversal through endothelial cells to exit the blood stream as well as the entering into the target cell.

As aforementioned, for a functional and reliable oral drug delivery via nanocarriers, the crossing of multiple cellular borders is crucial. The barrier function of epithelial cells is achieved by a cell connection via tight junctions, adherens junctions, and desmosomes. Together, this results in nearly no intercellular space between the cells. In endothelial complexes, these properties are accomplished by tight junctions, adherens junctions, and gap junctions (Bazzoni & Dejana, 2004). Epithelial cells cover nearly all tissues, whereas endothelial cells are only present on the interior surface of blood and lymphatic vessels. The biological function of both is a protection mechanism of the underlying tissue. Although being diverse in their morphology, virtually all endo- and epithelial cells are polarized and consist of an apical and a basal side. One of the most challenging aspects of drug delivery via nanocarriers is to overcome these barriers. Therefore, a fundamental understanding of the molecular mechanisms of entering and crossing epi- and endothelial cells is crucial for the development of nanomedicines.

In this review, we focus on the first barrier of the human body for nanocarriers to overcome the intestinal barrier. The complex structure of the human gastrointestinal tract is established by villi, which increase the absorptive intestinal surface area to about 300–400 m^2^ (Schenk & Mueller, 2008). Enterocytes, goblet cells, and M cells, all linked via tight junctions, represent the majority of cells in a villus. The small intestine, naturally responsible for the absorption of nutrients, is divided into duodenum, jejunum, and ileum. In these areas, villi are covered with mucus layers with a different thickness, ranging from 120 µm to 480 µm (Ensign et al., 2012). Mucus is produced by goblet cells to protect epithelial cells from bacterial interactions as well as physical damage by ingested food (Kim & Ho, 2010). However, despite being unarguably highly important for the protection of the human intestine, it marks the very first hurdle to overcome for orally applied nanocarriers. Many nanomaterials get immobilized by the mucus gel layer (Olmsted et al., 2001) and thus are not even able to reach the intestinal epithelial cell layer. Especially a decoration with polyethylene glycol (PEG) chains (Lai et al., 2007; Wang et al., 2008), the combination of anionic and cationic charges (Lai et al., 2009; de Sousa et al., 2015) and the usage of self-nanoemulsifying drug delivery systems (SNEDDS) (Hintzen et al., 2014) are valuable approaches to penetrate the mucosal barrier (Dünnhaupt et al., 2015). A different approach is the decoration of NPs with proteolytic enzymes like papain to cleave mucoglycoprotein substructures, which demonstrably increases mucosal penetration (Müller et al., 2013).

The major obstacle after penetrating the mucus layer is, however, crossing the first line of epithelial cells. Once the nanocarrier reaches the apical side of the cells, there are two possibilities for the transport to the basal side. The first is a paracellular transport, which involves a loosening of tight junctions and a transport between epithelial cells without a cellular uptake. A promising material for achieving paracellular transport is chitosan, a polysaccharide, which is demonstrably able to reversibly open tight junctions (Rosenthal et al., 2012). Nevertheless, for most nanocarrier systems, a paracellular transport is either toxic when inevitably also other constituents of the feces are diffusing through the opened paracellular route or simply not feasible due to size restrictions. The second possibility is transcytosis, which is defined as the transport of a molecule through the interior of a cell. This process consists of an uptake, preferably endocytosis, a transport within the cell as well as a withdrawal from the interior of the cell, namely exocytosis.

## Lysosomes as final fate for nanocarriers

Lysosomes are spherical, ovoid, or tubular intracellular vesicles with an acidic pH value (pH 4.5–5) (Mindell, 2012). Their size varies between <1 µm and several microns, depending on the cell type (Saftig, 2005). The physiologic purpose is mainly an enzymatic degradation or a respective recycling of either foreign molecules or cellular compounds (Saftig & Klumperman, 2009). In addition, lysosomes are demonstrably involved in secretion, plasma membrane repair, signaling, and energy metabolism processes (Settembre et al., 2013). Despite being an absolute necessity for eukaryotic cells, the lysosomal system marks one of the biggest hurdles for the transcytosis of nanocarriers. Regarding the majority of nanocarrier systems, the tendency can be described as EEDD: easy entrance, difficult discharge. This implies that the process of endocytosis is substantially easier than exocytosis for nanocarriers. Once NPs get taken up by cells, an overwhelming majority gets trafficked along the endolysosomal pathway. This typically involves a transport from early to late endosomes and their maturation into or fusion with lysosomes (Hofmann et al., 2014; Hu et al., 2015; Lerch et al., 2015). If a NP reaches this ‘final destination’ of a eukaryotic cell, there is often no escape. This means the carrier either gets degraded via enzymes or is, depending on the material, infinitely accumulated. This especially, however not exclusively, applies for the trafficking through the epithelial cell layer in the small intestine, since it is the first barrier to overcome for nanocarrier systems.

**Figure 2.**
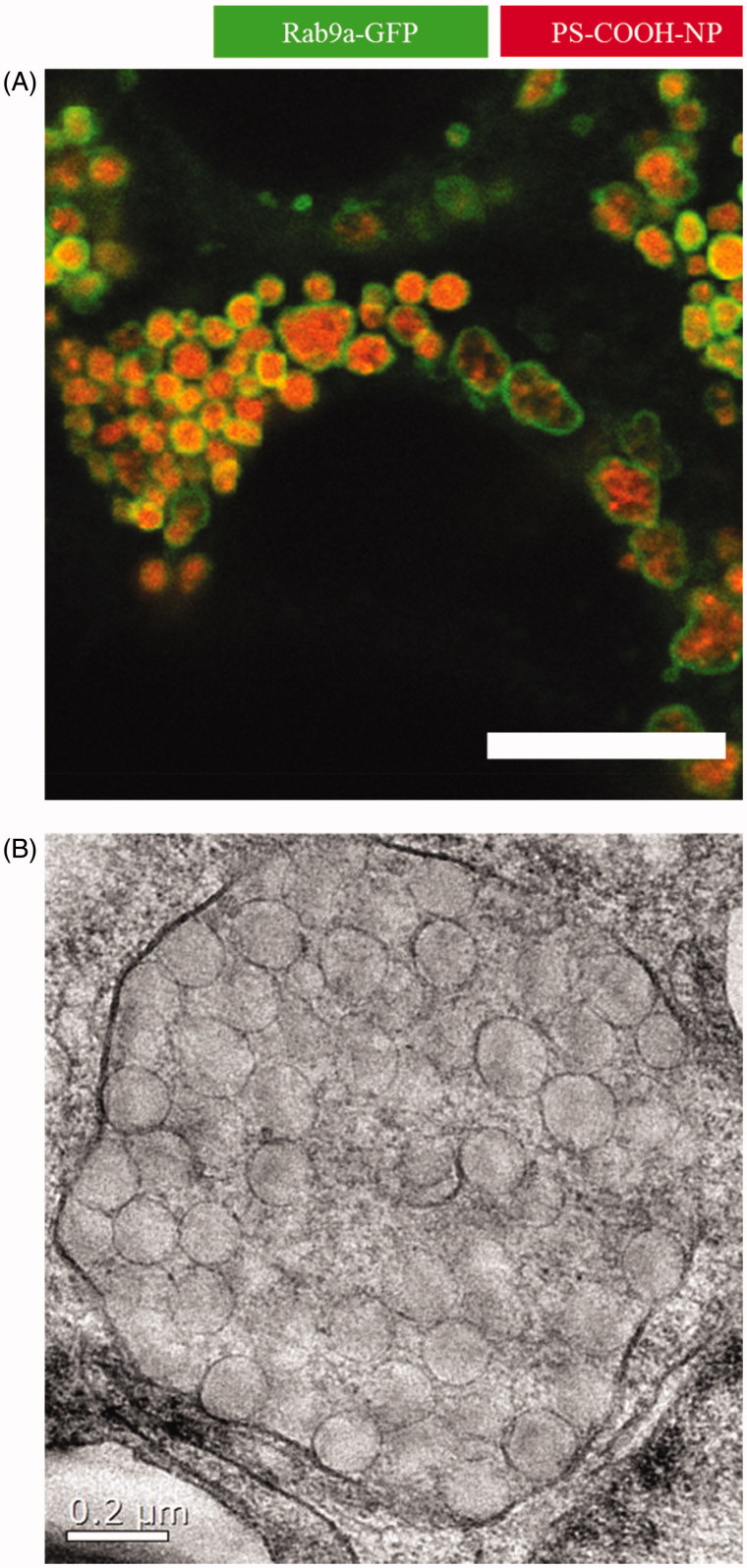
Close-up of nanoparticles trapped in late endosomes/lysosomes. In this study, Caco-2 cells were incubated with 500 µg/mL polystyrene nanoparticles for 24 hours. The addition resulted in huge endolysosomal structures up to 2 µm in diameter as analyzed by (A) confocal laser scanning microscopy (scale bar =10 µm) and (B) transmission electron microscopy (Reinholz et al., 2018). Reprinted from J. Reinholz, C. Diesler, S. Schöttler, M. Kokkinopoulou, S. Ritz, K. Landfester, V. Mailänder, Protein Machineries defining Pathways of Nanocarrier Exocytosis and Transcytosis, Acta Biomaterialia, Copyright (2018) with permission from Elsevier.

Figure 2 shows an example for nanocarriers stuck in the endolysosomal system. Here, polystyrene NPs in a concentration of 500 µg/mL were added to Caco-2 cells and incubated for 24 hours. This resulted in huge endolysosomal structures up to 2 µm in diameter as revealed by cLSM and TEM studies. In order to avoid such an accumulation or degradation, major development in the field of nanocarrier synthesis has to be done. Either the endolysosomal needs to be avoided completely or endosomes or lysosomes need to be triggered to fuse with the cell membrane and release the nanocarriers or nanocarriers need to escape the endolysosomal system to be able to get released from the cell again.

Next, we highlight different mechanisms for uptake, transcytosis, and exocytosis and discuss the effects of different nanocarrier properties and functionalizations.

## Endocytosis in epithelial and endothelial cells

In general, endo- and epithelial cells use multiple pathways of internalization. Endocytosis mechanisms and efficiencies mainly depend on size, charge, and surface properties of the nanomaterial. The major pathways for the internalization of nanomaterials in endo- and epithelial cells are clathrin-mediated, caveolin-mediated, and lipid-raft-mediated endocytosis as well as macropinocytosis, phagocytosis, and receptor-mediated endocytosis (Sahay et al., 2010). For the determination of endocytosis pathways, often pharmacological inhibitors are used (Iversen et al., 2011; Dutta & Donaldson, 2012). For example, chlorpromazine can inhibit clathrin-mediated uptake by a inducing a reversible translocation of clathrin and adapter proteins from the plasma membrane to vesicles (Wang et al., 1993); amiloride and EIPA inhibit micropinocytosis by either lowering the submembranous pH value and preventing Rac1 and Cdc42 signaling (amiloride) or inhibiting the Na^+^/H^+^ pump on plasma membranes (EIPA) (Kerr & Teasdale, 2009; Koivusalo et al., 2010; Ozdener et al., 2016); genistein can inhibit caveolae-mediated uptake via its function as a tyrosine kinase inhibitor (Pelkmans et al., 2002); and methyl-β-cyclodextrin depletes cholesterol from the cell membrane and can therefore also be used for the inhibition of caveolae-mediated uptake (Mahammad & Parmryd, 2015). However, nearly all pharmacological inhibitors induce side effects and can effect multiple pathways of internalization (Iversen et al., 2011). Many studies focus on endocytosis in Caco-2 (human colorectal adenocarcinoma) cells, since the intestine is the first barrier to overcome within the scope of oral drug delivery via nanocarriers. In general, positively charged NPs tend to be taken up better than negatively charged NP, which could be shown for 50 nm and 100 nm PS-NP in Caco-2 cells (Bannunah et al., 2014). The same study pointed out that positively charged NPs were endocytosed via a mixture of the clathrin-mediated pathway, a cholesterol-dependent pathway, and micropinocytosis, while negatively charged NP uptake was primarily mediated via lipid raft pathway (caveolae). Endocytosis of exogenous iron-loaded ferritin was shown to be clathrin-mediated (Antileo et al., 2013), while for 80 nm PLGA-NPs, a co-mediation of clathrin, lipid raft, caveolin, and macropinocytosis was observed (He et al., 2013). Wheat germ agglutinin-functionalized polymeric NPs with a size around 120 nm were also shown to be predominantly internalized via the clathrin-mediated pathway (Song et al., 2012). In addition, some studies examined the endocytosis pathways of natural material in Caco-2 cells. For example, pure DNA fragments as well as human IgG are presumably internalized via macropinocytosis (Sato et al., 2009; Johannessen et al., 2013), whereas for vitamin B_12_, the clathrin-mediated pathway is used (Fowler et al., 2013). However, by combining such natural material with NPs via bioconjugations, the endocytosis mechanisms can be altered, which can subsequently be used to increase the uptake and possibly the transcytosis efficiency.

A second cell line widely used in the investigation of endocytosis in epithelial cells is the MDCK cell line (Madin-Darby canine kidney epithelial cells). Recent studies show that 100 nm PLGA-NPs in these cells are internalized via caveolae/lipid-raft-mediated endocytosis as well as via clathrin-mediated endocytosis, while no macropinocytosis was observed (He et al., 2013). On the other hand, positively charged 20 nm and 120 nm PS-NPs enter MDCK cells predominantly via clathrin-mediated endocytosis (Fazlollahi et al., 2011). The PEG-PLA NPs with a size of approximately 90 nm were internalized mainly via clathrin-mediated endocytosis, while macropinocytosis was also involved (Harush-Frenkel et al., 2008). Furthermore, positively charged hexanoyl-chitosan NPs entered MDCK cells far more efficiently than negatively charged succinyl-chitosan NPs (Zubareva et al., 2015).

In contrast to epithelial cells, caveolae are believed to be the most abundant endocytotic structures in endothelial cells lining the blood vessels (Ehrenberg et al., 2009). Thus, many studies have focused on caveolin-mediated endocytosis of nanomaterial. For example, 20–100 nm BSA-coated polymeric NPs in BLMVEC (bovine lung microvascular endothelial) cells mainly get internalized via this pathway (Wang et al., 2009). Approximately 70 nm sized nano-Mg(OH)_2_ particles are endocytosed via the same caveolin-mediated pathway in HUVEC (human umbilical vein endothelial) cells (Meng et al., 2015). However, for 43 nm PS-NP in HUVEC cells, a clathrin-mediated uptake was shown. Interestingly, the same cells endocytosed 24 nm PS-NP via a clathrin- and caveolin-independent mechanism (Lai et al., 2007). Similar results were shown for 20 nm and 40 nm gold NPs in HMEC-1 (human microvascular endothelial) cells, although in this study also, macropinocytosis was observed (Landgraf et al., 2015). Moreover, like in epithelial cells, different functionalizations of NPs also lead to an altering of pathways. For example, 35 nm poly(2-hydroxypropylmethacrylamide)-coated gold NPs were internalized into hCMEC/D3 (human brain endothelial) cells by a clathrin- and caveolin-independent mechanism involving flotillin (Freese et al., 2013).

The adsorption of proteins on nanomaterial could potentially play a role in endothelial and epithelial endocytosis processes. This is widely discussed for the intravenous route of application but not for the oral route. Once a nanocarrier comes in contact to body fluids (in case of oral drug delivery possibly already in the mouth and GI tract, and in case of intravenous application in the blood stream), it gets covered with proteins (Cedervall et al., 2007; Walczyk et al., 2010; Schöttler et al., 2016). This forms a so-called protein corona and can substantially alter endocytosis efficiencies, as described for other cell lines like HeLa cells or human mesenchymal stem cells (Ritz et al., 2015) and might therefore also alter cellular pathways. Next, no research has been done about utilizing these differences in protein corona compositions regarding transcytosis processes in endo- or epithelial cells. To our knowledge, the only study so far that focused on the utilization of a protein corona for transcytosis reported differences in transcytosis efficiencies after the formation of a protein corona in HUVECs; however, these effects varied widely between different sized NPs (Ho et al., 2018). The identification of protein–NP interactions in the intestine and the implications for transcytosis might therefore be one of the new and most fascinating challenges for the development of nanocarriers for oral drug delivery.

In summary, pathways of endocytosis in both endo- and epithelial cells show remarkable similarities (Table 1). Recent results show that both cell types use more than one pathway for the internalization of (nano)-material. However, clathrin-mediated endocytosis and caveolin-mediated endocytosis seem to play a key role in most of the examined nanomaterial and cell lines. The choice of nanocarriers and their respective sizes and charges therefore needs to be thoroughly evaluated. Although thought to be important, there is not much work done in the field of connecting uptake mechanisms into gut endothelial cell lines and correlating it with transcytosis efficiency. Here, we see an important field for further research.

**Table 1.** Uptake mechanisms of various nanomaterials in endo- and epithelial cell lines.

NP characterization	NP size	Uptake mechanism	Strategy	Cell line	Study
Positively charged PS-NP	50 and 100 nm	Clathrin-mediated, cholesterol-dependent, and macropinocytosis	Chlorpromazine, amiloride Methyl-β-cyclodextrin	Caco-2	(Bannunah et al., 2014)
Negatively charged PS-NP	50 and 100 nm	Lipid-raft-associated uptake (caveolae)	Genistein	Caco-2	(Bannunah et al., 2014)
PLGA-NP	80 nm	Co-mediation of clathrin, lipid raft/caveolae, and macropinocytosis	Methyl-β-cyclodextrin, EIPA, PAO, nystatin, indomethacin	Caco-2	(He et al., 2013)
WGA-PEG-NP	120 nm	Clathrin-mediated, depending on surface PEG length	Chlorpromazine	Caco-2	(Song et al., 2012)
PLGA-NP	100 nm	Clathrin-mediated, lipid-raft-mediated	Nystatin, hyperosmotic sucrose	MDCK	(He et al., 2013)
PS-NP	20 and 120 nm	Clathrin-mediated	Monodansylcadaverine for cLSM studies	MDCK	(Fazlollahi et al., 2011)
Positively charged PEG-PLGA	90 nm	Clathrin-mediated, partially macropinocytosis	Dominant-negative mutant polypeptides of clathrin, cLSM analysis	MDCK	(Harush-Frenkel et al., 2008)
Negatively charged PEG-PLGA	96 nm	Clathrin-mediated, partially macropinocytosis	Dominant-negative mutant polypeptides of clathrin, cLSM analysis	MDCK	(Harush-Frenkel et al., 2008)
BSA-coated NP	20, 40, and 100 nm	Caveolae-mediated	cLSM analysis	BLMVEC	(Wang et al., 2009)
Nano-Mg(OH)_2_ particles	70 nm	Caveolae-mediated	cLSM analysis	HUVEC	(Meng et al., 2015)
COOH-PS-NP	43 nm	Clathrin-mediated	Chlorpromazine	HUVEC	(Lai et al., 2007)
COOH-PS-NP	24 nm	Cholesterol-independent, non-clathrin-mediated, and non-caveolae-mediated	Various inhibitors	HUVEC	(Lai et al., 2007)
Gold NP	20 nm	Caveolae-mediated	Genistein	HMEC-1	(Landgraf et al., 2015)
Gold NP	40 nm	Clathrin-mediated and macropinocytosis	Chlorpromazine, wortmannin	HMEC-1	(Landgraf et al., 2015)
Gold NP	35 nm	Flotillin-dependent	cLSM analysis	hCMEC/D3	(Freese et al., 2013)

## Transcytosis of nanomaterials

For most applications, nanocarriers need to enter and cross a layer of endo- and/or epithelial cells in order to successfully deliver their cargo. While there is the possibility of a paracellular transport, the key mechanism of overcoming these natural barriers is transcytosis. Nanocarriers designed for oral drug delivery at one point need to cross the intestinal barrier before being able to reach their target cells. Intravenous applied nanocarriers on the other hand need to cross the respective endothelial cell layers, for example the blood–brain barrier, to escape from the blood stream. However, the transcytosis efficiency of nanomaterial in these cells is fairly low, which limits the therapeutic possibilities for nanomedicine. It is thus essential to understand the molecular mechanisms behind transcytosis of nanomaterials.

The most common tool for transcytosis experiments is the so-called transwell system. Epithelial or endothelial cells are grown on a porous membrane to reach confluence and final differentiation. The barrier function can be tested by measuring the electric resistance of the monolayer, which is called TEER value (Ω × cm^2^). The transwell chamber itself consists of an apical and a basal side. If nanomaterial is added into the apical medium, the subsequently transcytosed fraction can be measured in the basal medium.

A range of studies focused on the transcytosis efficiency of different nanomaterials in different cell types. Interestingly, nearly all of them showed that only a minority of NPs is actually transcytosed through epi- and endothelial cell layers *in vitro* as well as *in vivo*. While some nanomaterials are simply not sufficiently taken up, the major problem for this lack of efficiency seems to be the fact that the majority of nanomaterials, after endocytosis, is trafficked within the endolysosomal pathway (He et al., 2013; Ye et al., 2013). Thus, the final fate of this pathway is the storage or degradation of the respective nanomaterial in lysosomes.

While transcytosis in general is a partially energy-dependent process (He et al., 2013), the efficiency is largely impacted by size and charge of the examined nanomaterial. The smaller the NP, the better it is transcytosed across, for example through the intestine (Fowler et al., 2013) or the blood–brain barrier (Liu et al., 2014). Regarding the charge of nanomaterial, most but not all studies point out that positively charged polymeric NPs tend to cross epithelial cell layers with a higher efficiency compared to negatively charged NP. This could, for example, be shown for amidine- and carboxyl-modified 20–120 nm sized PNP in MDCK cells (Fazlollahi et al., 2011). A significantly higher NP flux was also observed for aminated latex particles compared to their carboxylated equivalents in Caco-2 cells (des Rieux et al., 2005). This difference in transcytosis efficiency might be due to the fact that positively charged NPs are able to avoid the lysosomal system, while negatively charged NPs accumulate inside lysosomes (Harush-Frenkel et al., 2008).

A successful transcytosis event potentially includes trafficking routes via apical early endosomes (AEE), common endosomes and basolateral sorting endosomes (BSE) as well as sorting via the Golgi complex as shown by co-localization as well as inhibitor studies in MDCK cells (He et al., 2013). In addition to sorting via the Golgi complex, the endoplasmic reticulum might be involved in transcytosis, as postulated for Caco-2 cells (He et al., 2013). On the contrary, the transcytosis of albumin-coated NPs in BLMVEC cells was shown to be solely reliant on caveolae and caveosomes (Wang et al., 2009). Last but not least, the whole process of transcytosis might additionally be dependent on the regulation of cytoskeleton and motor proteins (Wang et al., 2008; He et al., 2013).

Recent research additionally focused on improving the rate of transcytosis via NP bioconjugations with different proteins. For example, a functionalization of polymeric NPs with the constant portion of IgG (Fc part of immunoglobulin G) significantly increased the rate of transcytosis in Calu-3 airway epithelial cells (Vllasaliu et al., 2012). In Caco-2 cells, transcytosis efficiency could be enhanced by 2-fold via this modification (Pridgen et al., 2013). The same study revealed that targeting of the FcRn receptor *in vivo* enhanced the absorption efficiency from 1.2% per hour to 13.7% per hour. In addition, such targeted NPs containing insulin were able to achieve a prolonged hypoglycemic response in mice when orally administered at a clinically relevant insulin dose. The authors concluded that Fc-region-modified NPs essentially avoid the lysosomal pathway and are subsequently transcytosed with a higher efficiency. Similarly, a bioconjugation with vitamin B_12_ of 50 nm or 100 nm PS-NP not only increased the transport by 3-fold (for 50 nm NP) and 7-fold (100 nm), but also led to a pathway switch and prevented the NP trafficking to lysosomes (Fowler et al., 2013). Yet another approach for oral drug delivery is a surface functionalization with folic acid to target the folate receptor. PLGA-NPs modified with folic acid highly increased the transcytosis efficiency in Caco-2 cells and might be used to enhance oral bioavailability (Roger et al., 2012).

**Figure 3.**
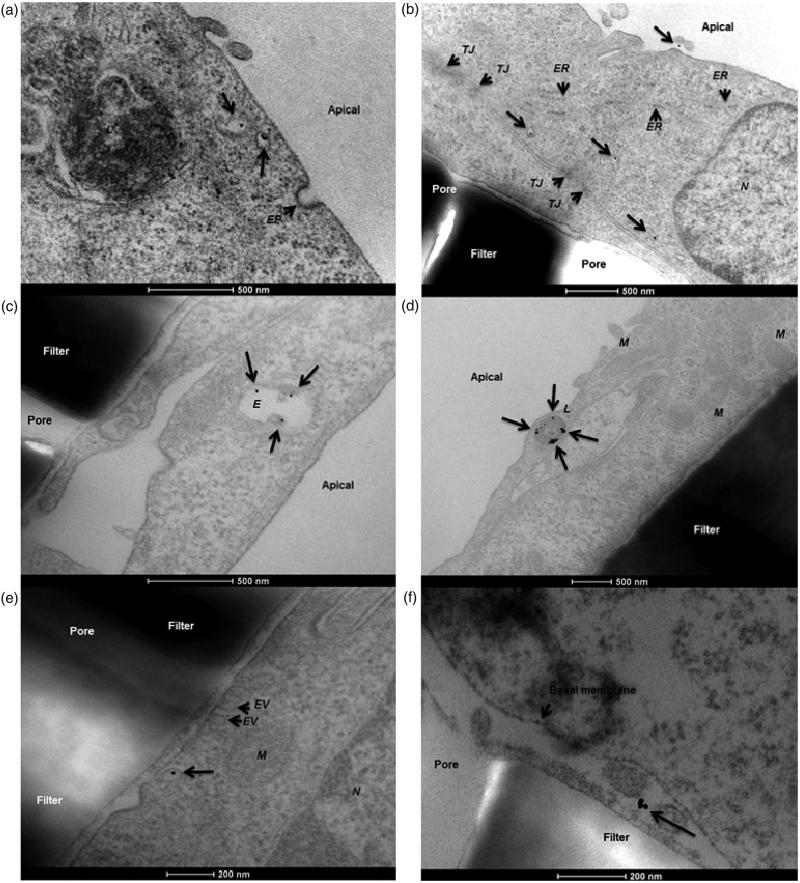
Transcytosis of 12 nm human serum albumin–gold nanoparticles across a hCMEC/D3 endothelial cell monolayer. In this study, the authors demonstrated a protocol for the *in vitro* determination of nanoparticle translocation across a cell monolayer using transmission electron microscopy. (a) Nanoparticles present in apical sorting vesicles within the cytosolic space. (b) Gold nanoparticles detected in different cytoplasmic regions. (c) Nanoparticles co-localizing with endosomes. (d) Nanoparticles co-localizing with lysosomes. (e) A single gold nanoparticle exits the basolateral cell membrane from a vesicle. (f) The nanoparticle is finally present in the basal compartment. EP: endocytic pit; ER: endoplasmic reticulum; EV: exocytic vesicle; E: endosome; L: lysosome; M: mitochondria; N: nucleus; TJ: tight junction (Ye et al., 2015). Reproduced from D. Ye, K.A. Dawson, I. Lynch, A TEM protocol for quality assurance of *in vitro* cellular barrier models and its application to the assessment of nanoparticle transport mechanisms across barriers, Analyst 140(1) (2015) 83-97 with permission of The Royal Society of Chemistry.

On the contrary, NPs targeted to the brain can be functionalized with transferrin to cross the blood–brain barrier more efficiently via transferrin receptor-mediated transcytosis, which could be shown for 45 and 80 nm sized NPs (Wiley et al., 2013). A different study revealed that coating iron oxide NPs with PECAM-1 antibodies also considerably enhances the NP flux across hCMEC/D3 cells, another model for the blood–brain barrier (Dan et al., 2013). An example for transcytosis of 12 nm gold NPs across a hCMEC/D3 endothelial cell layer grown on transwells observed via TEM can be found in Figure 3 (Ye et al., 2015).

Additionally, a promising candidate, respectively, target for the intestinal transcytosis of nanocarriers might be M (microfold) cells, a cell type which is mainly localized in Peyer’s patches in the small intestine (Gebert et al., 1996). Their natural function is the transcytosis of antigens across the gut epithelium (Mabbott et al., 2013). One of the major advantages of this cell type is the fact that the mucus layer is considerably thinner and thus cells are easier to access (Frey et al., 1996). M cells in general are reported to have high transcytotic activities in apical-to-basal direction (Gebert et al., 2004; Fievez et al., 2009). In addition, M cells feature a significantly reduced intracellular lysosomal activity (Kunisawa et al., 2012). The transcytosis of nanocarriers through M cells was already reported for several nanomaterials (Clark et al., 2000; Parayath et al., 2015) and M cells can also be targeted directly via nanocarrier surface modifications. For example, PEGylated PLGA-based NPs featuring RGD peptides as a targeting ligand at their surface were used to successfully target the β1 integrins on the apical side of M cells (Garinot et al., 2007).

In summary, many studies have dealt with the process of transcytosis. Many different NP and cell combinations have been examined and the molecular mechanisms as well as the rate of transcytosis strongly depend on the utilized nanomaterial and its surface functionalization with targeting molecules. However, a reliable therapeutic nanocarrier system either for oral drug delivery or for crossing the blood–brain barrier still requires an improved transcytosis efficiency.

## Exocytosis of nanomaterials

After nanomaterials enter animal cells via one of the endocytosis mechanisms outlined above, the cell determines the further trafficking. While it is possible that the nanocarrier gets: (1) degraded in lysosomes, (2) trafficked to a specific cellular organelle, or (3) released its cargo inside the cytosol, the cell might also (d) traffic it to the extracellular space. This process is called exocytosis and is of high interest due to the fact that nanocarriers need to be released from the cell to successfully transverse a barrier.

In general, exocytosis is an energy-dependent process, which could be shown for poly(d,l-lactide-co-glycolide) NPs with a size of approximately 100 nm in vascular smooth muscle cells (Panyam & Labhasetwar, 2003). In addition, the size of nanomaterial seems to have a keen impact on the rate of exocytosis. In Hela cells, transferrin-coated Au NPs with sizes ranging from 14 to 100 nm were compared. The study revealed that smaller NPs were exocytosed at a faster rate and at a higher overall percentage than larger NPs (Chithrani & Chan, 2007). Furthermore, the exocytosis rate of silicon particles loaded with 15 nm and 30 nm SPIONs in macrophages was examined via iron measurement of the cell culture media 2–7 days after the NP treatment. A significantly higher amount of iron was found for 15 nm SPIONs, indicating a higher efficiency of exocytosis for the smaller NPs (Serda et al., 2010). Apparently, the surface chemistry of NPs also appears to affect the exocytosis efficiency, which could be shown for macrophages treated with gold NPs. While PEGylated gold NPs were exocytosed rather rapidly, cationic gold NPs agglomerated inside the cell, which significantly delayed their exocytosis (Oh & Park, 2014). The authors therefore concluded that exocytosis, at least for gold NPs in macrophages, can be manipulated via surface modifications.

Exocytosis events of NPs are rather rare, which contributes to the low efficiency of transcytosis. Primarily depending on the endocytosis mechanism, several exocytosis pathways are possible: lysosomal escape and subsequent exocytosis, lysosomal fusion with the plasma membrane, multivesicular body (MVB) or late endosome fusion with the plasma membrane, and fusion of caveolae or caveosomes with the plasma membrane (Wang et al., 2011; Sakhtianchi et al., 2013). In accordance with the notable variability of endocytotic pathways, different nanomaterials are released to the extracellular space via different exocytosis mechanisms in different cell types.

In general, lysosomal fusion with the plasma membrane is a Ca^2+^-regulated process which is *inter alia* necessary for membrane repair mechanisms (Gerasimenko et al., 2001). An example for lysosomal fusion with the plasma membrane with subsequent exocytosis could be shown for phosphonate-modified mesoporous silica NPs with an approximate size of 130 nm in various cell lines. Within 24 h, A549 (lung cancer) cells showed the highest exocytosis rate, followed by MDA-MB 231 (breast cancer), PANC-1 (pancreatic cancer), MCF-7 (breast cancer), MDA-MB 435 (melanoma cancer), and H9 (human embryonic stem cell line) cells. The lysosomal fusion and secretion were shown via cLSM co-localization studies and via measurements of the enzyme β-hexosaminidase, a marker for lysosomal exocytosis (Yanes et al., 2013). Moreover, the study revealed that the Golgi apparatus was not involved in exocytosis in any of the tested cell lines. However, pharmacological inhibitors for microtubule formation and actin polymerization both inhibited exocytosis, proposing a function of both for the lysosomal transport and plasma membrane fusion, as already stated by previous research (Cordonnier et al., 2001). A different study focused on 50 nm sized silica NPs in H1299 (human lung carcinoma) cells. The study revealed that NP clusters in lysosomes were easily exocytosed in comparison with free NPs in the cytoplasm (Chu et al., 2011).

In addition to lysosomal exocytosis, exocytosis of MVBs might play a key role in the secretion of nanomaterial. TEM images of SPIONs trafficked in J774 macrophages illustrated a possible fusion of MVBs with the plasma membrane, which might be the mechanism responsible for the NP secretion (Serda et al., 2010). Similar results proposing a possible key role for MVB in SPION secretion were observed in HMVEC (human microvascular vein endothelial) cells (Ferrati et al., 2010). A study examining sub-100 nm Au-NP in Hela cells showed that NPs after uptake were trafficked toward the plasma membrane in late endosomes and lysosomes. The vesicles fused with the membrane and subsequently released the NPs, which were finally secreted by the cells (Chithrani & Chan, 2007).

On the other hand, lipid rafts and especially cholesterol might also be involved in the process of nanomaterial exocytosis (Salaun et al., 2004). A study focusing on the uptake of 60 nm sized cationic polysaccharide NPs in 16HBE cells observed a considerable increase in endocytosis after cholesterol depletion. To further analyze this result, they added filipin into the medium of NP-treated cells, which significantly decreased the rate of exocytosis (Dombu et al., 2010). Finally, exocytosis can also be triggered by increasing the extracellular calcium concentration (Oheim et al., 2006). This phenomenon was examined for gold NPs in HT-29 (human colonic adenocarcinoma) cells. It could be revealed that different Ca^2+^ concentrations in the medium (0–10 mM) influence the amount of exocytosed NPs. While the physiological extracellular calcium concentration in mammals is roughly 2 mM, even higher concentrations up to 10 mM increased the amount of exocytosed gold NPs (Chen et al., 2010).

**Figure 4.**
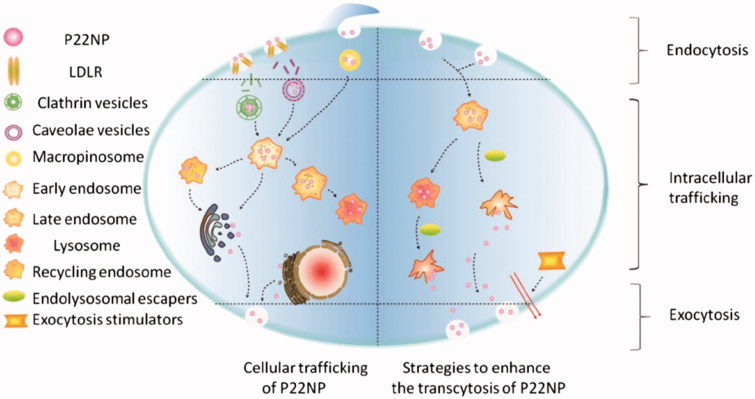
Putative pathways for endolysosomal escape and exocytosis of P22NPs in Caco-2 cells. The addition of hemagglutinin-2 and metformin led to an increase in endolysosomal escape and exocytosis of nanoparticles, overall resulting in an increase in transcytosis. The black arrows represent the pathways that were demonstrated by the authors (Cui et al., 2018). Reproduced from Y. Cui, W. Shan, R. Zhou, M. Liu, L. Wu, Q. Guo, Y. Zheng, J. Wu, Y. Huang, The combination of endolysosomal escape and basolateral stimulation to overcome the difficulties of ‘easy uptake hard transcytosis’ of ligand-modified nanoparticles in oral drug delivery, Nanoscale (2018) with permission of The Royal Society of Chemistry.

Most recently, a study shed light into potential endolysosomal escape and the resulting positive increase for exocytosis and a hereby associated increased transcytosis efficiency. Here, the addition of hemagglutinin-2 and/or metformin resulted in an increase in endolysosomal escape and subsequent exocytosis of P22NPs (NH_2_-C6-[cMPRLRGC]c-NH_2_ NPs). By simultaneously adding these two chemicals, the combination of endolysosomal escape and exocytosis triggered an increase in transcytosis efficiency of encapsulated insulin versus free insulin by a factor up to 5.1 (Cui et al., 2018). The suspected pathway for successful combination of endolysosomal escape and basolateral stimulation is depicted in Figure 4.

In essence, exocytosis is a very diverse and complex process with a variety of different factors involved. For many applications, especially drug delivery through natural barriers via nanocarriers, an exocytosis event is crucial at least once. The pathways show a huge diversity, depending on the material, surface chemistry, charge, size, and of course the different mammalian cell lines. A deeper understanding of the exocytosis process in epi- and endothelial cells might in addition contribute to an enhancement in transcytosis efficiencies. Thus, the process of exocytosis needs to be further examined in a broader spectrum of nanomaterials and cell lines.

## Conclusion

In theory, oral drug delivery via nanocarriers marks one if not the most promising approach for the oral delivery of pharmaceuticals. At least for peptide-based drugs, such a carrier system is urgently needed for a protection against the proteolytic environment in the stomach. However, several natural barriers of the human body impede the uptake or cellular traversal of nanocarrier systems. In the future, further research and development are needed to solve the following main problems of oral drug delivery via nanocarriers: (1) an enhanced penetration of the intestinal mucus barrier, (2) reliable uptake by enterocytes or other intestinal cell types, (3) enhanced efficiencies for the transversal of the intestinal cell barrier, in particular a solution for the often problematic trafficking into the endolysosomal system, and (4) a second transversal of endothelial cells to finally reach the bloodstream. Once these problems are solved, the encapsulation or complexation of a huge variety of drugs into nanocarriers could replace the intravenous injection of the pure drug, making millions of people’s daily life easier and offer hitherto undescribed possibilities.

## References

[CIT0001] According to personal communication from abdata.de (2017)

[CIT0002] Anselmo AC, Mitragotri S (2016). Nanoparticles in the clinic. Bioeng Transl Med 1:10–29. 29313004 10.1002/btm2.10003 PMC5689513

[CIT0003] Antileo E, Garri C, Tapia V, et al. (2013). Endocytic pathway of exogenous iron-loaded ferritin in intestinal epithelial (Caco-2) cells. Am J Physiol Gastrointest Liver Physiol 304:G655–61. 23370673 10.1152/ajpgi.00472.2012

[CIT0004] Bannunah AM, Vllasaliu D, Lord J, Stolnik S (2014). Mechanisms of nanoparticle internalization and transport across an intestinal epithelial cell model: effect of size and surface charge. Mol Pharmaceutics 11:4363–73. 10.1021/mp500439c 25327847

[CIT0005] Bazzoni G, Dejana E (2004). Endothelial cell-to-cell junctions: molecular organization and role in vascular homeostasis. Physiol Rev 84:869–901. 15269339 10.1152/physrev.00035.2003

[CIT0006] Bobo D, Robinson KJ, Islam J, et al. (2016). Nanoparticle-based medicines: a review of FDA-approved materials and clinical trials to date. Pharm Res 33:2373–87. 27299311 10.1007/s11095-016-1958-5

[CIT0007] Cedervall T, Lynch I, Lindman S, et al. (2007). Understanding the nanoparticle–protein corona using methods to quantify exchange rates and affinities of proteins for nanoparticles. Proc Natl Acad Sci 104:2050–5. 17267609 10.1073/pnas.0608582104 PMC1892985

[CIT0008] Chen R, Huang G, Ke PC (2010). Calcium-enhanced exocytosis of gold nanoparticles. Appl Phys Lett 97:093706.

[CIT0009] Chithrani BD, Chan WC (2007). Elucidating the mechanism of cellular uptake and removal of protein-coated gold nanoparticles of different sizes and shapes. Nano Lett 7:1542–50. 17465586 10.1021/nl070363y

[CIT0010] Chu Z, Huang Y, Tao Q, Li Q (2011). Cellular uptake, evolution, and excretion of silica nanoparticles in human cells. Nanoscale 3:3291. 21743927 10.1039/c1nr10499c

[CIT0011] Clark MA, Hirst BH, Jepson MA (2000). Lectin-mediated mucosal delivery of drugs and microparticles. Adv Drug Deliv Rev 43:207–23. 10967227 10.1016/s0169-409x(00)00070-3

[CIT0012] Cordonnier MN, Dauzonne D, Louvard D, Coudrier E (2001). Actin filaments and myosin I alpha cooperate with microtubules for the movement of lysosomes. Mol Biol Cell 12:4013–29. 11739797 10.1091/mbc.12.12.4013 PMC60772

[CIT0013] Cui Y, Shan W, Zhou R, et al. (2018). The combination of endolysosomal escape and basolateral stimulation to overcome the difficulties of “easy uptake hard transcytosis” of ligand-modified nanoparticles in oral drug delivery. Nanoscale 10:1494–507. 29303184 10.1039/c7nr06063g

[CIT0014] Dan M, Cochran DB, Yokel RA, Dziubla TD (2013). Binding, transcytosis and biodistribution of anti-PECAM-1 iron oxide nanoparticles for brain-targeted delivery. PLoS One 8:e81051. 24278373 10.1371/journal.pone.0081051 PMC3835573

[CIT0015] de Sousa IP, Steiner C, Schmutzler M, et al. (2015). Mucus permeating carriers: formulation and characterization of highly densely charged nanoparticles. Eur J Pharm Biopharm 97:273–9. 25576256 10.1016/j.ejpb.2014.12.024

[CIT0016] des Rieux A, Ragnarsson EG, Gullberg E, et al. (2005). Transport of nanoparticles across an *in vitro* model of the human intestinal follicle associated epithelium. Eur J Pharm Sci 25:455–65. 15946828 10.1016/j.ejps.2005.04.015

[CIT0017] Dombu CY, Kroubi M, Zibouche R, et al. (2010). Characterization of endocytosis and exocytosis of cationic nanoparticles in airway epithelium cells. Nanotechnology 21:355102. 20689164 10.1088/0957-4484/21/35/355102

[CIT0018] Dünnhaupt S, Kammona O, Waldner C, et al. (2015). Nano-carrier systems: strategies to overcome the mucus gel barrier. Eur J Pharm Biopharm 96:447–53. 25712487 10.1016/j.ejpb.2015.01.022

[CIT0019] Dutta D, Donaldson JG (2012). Search for inhibitors of endocytosis: intended specificity and unintended consequences. Cell Logist 2:203–8. 23538558 10.4161/cl.23967 PMC3607622

[CIT0020] Ehrenberg MS, Friedman AE, Finkelstein JN, et al. (2009). The influence of protein adsorption on nanoparticle association with cultured endothelial cells. Biomaterials 30:603–10. 19012960 10.1016/j.biomaterials.2008.09.050

[CIT0021] Ensign LM, Cone R, Hanes J (2012). Oral drug delivery with polymeric nanoparticles: the gastrointestinal mucus barriers. Adv Drug Deliv Rev 64:557–70. 22212900 10.1016/j.addr.2011.12.009 PMC3322271

[CIT0022] Fazlollahi F, Angelow S, Yacobi NR, et al. (2011). Polystyrene nanoparticle trafficking across MDCK-II. Nanomedicine 7:588–94. 21310266 10.1016/j.nano.2011.01.008 PMC3130091

[CIT0023] Ferrati S, Mack A, Chiappini C, et al. (2010). Intracellular trafficking of silicon particles and logic-embedded vectors. Nanoscale 2:1512–20. 20820744 10.1039/c0nr00227e PMC2936484

[CIT0024] Fievez V, Plapied L, des Rieux A, et al. (2009). Targeting nanoparticles to M cells with non-peptidic ligands for oral vaccination. Eur J Pharm Biopharm 73:16–24. 19409989 10.1016/j.ejpb.2009.04.009

[CIT0025] Fonte P, Nogueira T, Gehm C, et al. (2011). Chitosan-coated solid lipid nanoparticles enhance the oral absorption of insulin. Drug Deliv Transl Res 1:299–308. 25788364 10.1007/s13346-011-0023-5

[CIT0026] Fowler R, Vllasaliu D, Trillo FF, et al. (2013). Nanoparticle transport in epithelial cells: pathway switching through bioconjugation. Small 9:3282–94. 23637086 10.1002/smll.201202623

[CIT0027] Freese C, Unger RE, Deller RC, et al. (2013). Uptake of poly(2-hydroxypropylmethacrylamide)-coated gold nanoparticles in microvascular endothelial cells and transport across the blood–brain barrier. Biomater Sci 1:824. 10.1039/c3bm60050e 32481928

[CIT0028] Frey A, Giannasca KT, Weltzin R, et al. (1996). Role of the glycocalyx in regulating access of microparticles to apical plasma membranes of intestinal epithelial cells: implications for microbial attachment and oral vaccine targeting. J Exp Med 184:1045–59. 9064322 10.1084/jem.184.3.1045 PMC2192803

[CIT0029] Garinot M, Fiévez V, Pourcelle V, et al. (2007). PEGylated PLGA-based nanoparticles targeting M cells for oral vaccination. J Control Release 120:195–204. 17586081 10.1016/j.jconrel.2007.04.021

[CIT0030] Gebert A, Rothkötter H-J, Pabst R (1996). M cells in Peyer's patches of the intestine. In: Kwang W. Jeon, ed. International review of cytology. San Diego, New York, Boston, London, Sydney, Tokyo, Toronto: Academic Press, 91–159. 10.1016/s0074-7696(08)61346-7 8768493

[CIT0031] Gebert A, Steinmetz I, Fassbender S, Wendlandt K-H (2004). Antigen transport into Peyer's patches: increased uptake by constant numbers of M cells. Am J Pathol 164:65–72. 14695320 10.1016/S0002-9440(10)63097-0 PMC1602236

[CIT0032] Gerasimenko JV, Gerasimenko OV, Petersen OH (2001). Membrane repair: Ca(2+)-elicited lysosomal exocytosis. Curr Biol 11:R971–4. 11728325 10.1016/s0960-9822(01)00577-2

[CIT0033] Harush-Frenkel O, Rozentur E, Benita S, Altschuler Y (2008). Surface charge of nanoparticles determines their endocytic and transcytotic pathway in polarized MDCK cells. Biomacromolecules 9:435–43. 18189360 10.1021/bm700535p

[CIT0034] He B, Jia Z, Du W, et al. (2013). The transport pathways of polymer nanoparticles in MDCK epithelial cells. Biomaterials 34:4309–26. 23478037 10.1016/j.biomaterials.2013.01.100

[CIT0035] He B, Lin P, Jia Z, et al. (2013). The transport mechanisms of polymer nanoparticles in Caco-2 epithelial cells. Biomaterials 34:6082–98. 23694903 10.1016/j.biomaterials.2013.04.053

[CIT0036] Hintzen F, Perera G, Hauptstein S, et al. (2014). *In vivo* evaluation of an oral self-microemulsifying drug delivery system (SMEDDS) for leuprorelin. Int J Pharm 472:20–6. 24879935 10.1016/j.ijpharm.2014.05.047

[CIT0037] Ho YT, Kamm RD, Kah JCY (2018). Influence of protein corona and caveolae-mediated endocytosis on nanoparticle uptake and transcytosis. Nanoscale 10:12386–12397. 10.1039/c8nr02393j 29926047

[CIT0038] Hofmann D, Tenzer S, Bannwarth MB, et al. (2014). Mass spectrometry and imaging analysis of nanoparticle-containing vesicles provide a mechanistic insight into cellular trafficking. ACS Nano 8:10077–88. 25244389 10.1021/nn502754c

[CIT0039] Hu Y-B, Dammer EB, Ren R-J, Wang G (2015). The endosomal-lysosomal system: from acidification and cargo sorting to neurodegeneration. Transl Neurodegener 4:18. 26448863 10.1186/s40035-015-0041-1 PMC4596472

[CIT0040] Iversen T-G, Skotland T, Sandvig K (2011). Endocytosis and intracellular transport of nanoparticles: present knowledge and need for future studies. Nano Today 6:176–85.

[CIT0041] Johannessen LE, Spilsberg B, Wiik-Nielsen CR, et al. (2013). DNA-fragments are transcytosed across CaCo-2 cells by adsorptive endocytosis and vesicular mediated transport. PLoS One 8:e56671. 23409196 10.1371/journal.pone.0056671 PMC3569430

[CIT0042] Kerr MC, Teasdale RD (2009). Defining macropinocytosis. Traffic 10:364–71. 19192253 10.1111/j.1600-0854.2009.00878.x

[CIT0043] Kim YS, Ho SB (2010). Intestinal goblet cells and mucins in health and disease: recent insights and progress. Curr Gastroenterol Rep 12:319–30. 20703838 10.1007/s11894-010-0131-2 PMC2933006

[CIT0044] Koivusalo M, Welch C, Hayashi H, et al. (2010). Amiloride inhibits macropinocytosis by lowering submembranous pH and preventing Rac1 and Cdc42 signaling. J Cell Biol 188:547–63. 20156964 10.1083/jcb.200908086 PMC2828922

[CIT0045] Kunisawa J, Kurashima Y, Kiyono H (2012). Gut-associated lymphoid tissues for the development of oral vaccines. Adv Drug Deliv Rev 64:523–30. 21827802 10.1016/j.addr.2011.07.003

[CIT0046] Lai SK, Hida K, Man ST, et al. (2007). Privileged delivery of polymer nanoparticles to the perinuclear region of live cells via a non-clathrin, non-degradative pathway. Biomaterials 28:2876–84. 17363053 10.1016/j.biomaterials.2007.02.021

[CIT0047] Lai SK, O'Hanlon DE, Harrold S, et al. (2007). Rapid transport of large polymeric nanoparticles in fresh undiluted human mucus. Proc Natl Acad Sci 104:1482–7. 17244708 10.1073/pnas.0608611104 PMC1785284

[CIT0048] Lai SK, Wang Y-Y, Hanes J (2009). Mucus-penetrating nanoparticles for drug and gene delivery to mucosal tissues. Adv Drug Deliv Rev 61:158–71. 19133304 10.1016/j.addr.2008.11.002 PMC2667119

[CIT0049] Landgraf L, Muller I, Ernst P, et al. (2015). Comparative evaluation of the impact on endothelial cells induced by different nanoparticle structures and functionalization. Beilstein J Nanotechnol 6:300–12. 25821668 10.3762/bjnano.6.28 PMC4362490

[CIT0050] Lerch S, Ritz S, Bley K, et al. (2015). Nanoprobing the acidification process during intracellular uptake and trafficking. Nanomedicine 11:1585–96. 25957068 10.1016/j.nano.2015.04.010

[CIT0051] Liu D, Lin B, Shao W, et al. (2014). *In vitro* and *in vivo* studies on the transport of PEGylated silica nanoparticles across the blood-brain barrier. ACS Appl Mater Interfaces 6:2131–6. 24417514 10.1021/am405219u

[CIT0052] Mabbott NA, Donaldson DS, Ohno H, et al. (2013). Microfold (M) cells: important immunosurveillance posts in the intestinal epithelium. Mucosal Immunol 6:666. 23695511 10.1038/mi.2013.30 PMC3686595

[CIT0053] Mahammad S, Parmryd I (2015). Cholesterol depletion using methyl-β-cyclodextrin. In: Dylan M. Owen, ed. Methods in membrane lipids. New York: Humana Press (part of Springer), 91–102. 10.1007/978-1-4939-1752-5_8 25331130

[CIT0054] Mei L, Zhang Z, Zhao L, et al. (2013). Pharmaceutical nanotechnology for oral delivery of anticancer drugs. Adv Drug Deliv Rev 65:880–90. 23220325 10.1016/j.addr.2012.11.005

[CIT0055] Meng N, Han L, Pan X, et al. (2015). Nano-Mg(OH)2-induced proliferation inhibition and dysfunction of human umbilical vein vascular endothelial cells through caveolin-1-mediated endocytosis. Cell Biol Toxicol 31:15–27. 25575676 10.1007/s10565-014-9291-4

[CIT0056] Mindell JA (2012). Lysosomal acidification mechanisms. Annu Rev Physiol 74:69–86. 22335796 10.1146/annurev-physiol-012110-142317

[CIT0057] Müller C, Leithner K, Hauptstein S, et al. (2013). Preparation and characterization of mucus-penetrating papain/poly (acrylic acid) nanoparticles for oral drug delivery applications. J Nanopart Res 15:1353.

[CIT0058] Oh N, Park JH (2014). Surface chemistry of gold nanoparticles mediates their exocytosis in macrophages. ACS Nano 8:6232–41. 24836308 10.1021/nn501668a

[CIT0059] Oheim M, Kirchhoff F, Stuhmer W (2006). Calcium microdomains in regulated exocytosis. Cell Calcium 40:423–39. 17067670 10.1016/j.ceca.2006.08.007

[CIT0060] Olmsted SS, Padgett JL, Yudin AI, et al. (2001). Diffusion of macromolecules and virus-like particles in human cervical mucus. Biophys J 81:1930–7. 11566767 10.1016/S0006-3495(01)75844-4 PMC1301668

[CIT0061] Ozdener GB, Bais MV, Trackman PC (2016). Determination of cell uptake pathways for tumor inhibitor lysyl oxidase propeptide. Mol Oncol 10:1–23. 26297052 10.1016/j.molonc.2015.07.005 PMC4549800

[CIT0062] Panyam J, Labhasetwar V (2003). Dynamics of endocytosis and exocytosis of poly(D,L-lactide-co-glycolide) nanoparticles in vascular smooth muscle cells. Pharm Res 20:212–20. 12636159 10.1023/a:1022219003551

[CIT0063] Parayath NN, Nehoff H, Müller P, et al. (2015). Styrene maleic acid micelles as a nanocarrier system for oral anticancer drug delivery–dual uptake through enterocytes and M-cells. Int J Nanomed 10:4653. 10.2147/IJN.S87681 PMC4516255 26229468

[CIT0064] Pelkmans L, Püntener D, Helenius A (2002). Local actin polymerization and dynamin recruitment in SV40-induced internalization of caveolae. Science 296:535–9. 11964480 10.1126/science.1069784

[CIT0065] Pridgen EM, Alexis F, Kuo TT, et al. (2013). Transepithelial transport of Fc-targeted nanoparticles by the neonatal fc receptor for oral delivery. Sci Transl Med 5:213ra167. 10.1126/scitranslmed.3007049 PMC4023672 24285486

[CIT0066] Reinholz J, Diesler C, Schöttler S, et al. (2018). Protein machineries defining pathways of nanocarrier exocytosis and transcytosis. Acta Biomater 71:432–443. 10.1016/j.actbio.2018.03.006 29530823

[CIT0067] Ritz S, Schöttler S, Kotman N, et al. (2015). Protein corona of nanoparticles: distinct proteins regulate the cellular uptake. Biomacromolecules 16:1311–21. 25794196 10.1021/acs.biomac.5b00108

[CIT0068] Roger E, Kalscheuer S, Kirtane A, et al. (2012). Folic acid functionalized nanoparticles for enhanced oral drug delivery. Mol Pharm 9:2103–10. 22670575 10.1021/mp2005388 PMC3461242

[CIT0069] Rosenthal R, Günzel D, Finger C, et al. (2012). The effect of chitosan on transcellular and paracellular mechanisms in the intestinal epithelial barrier. Biomaterials 33:2791–800. 22230222 10.1016/j.biomaterials.2011.12.034

[CIT0070] Saftig P, Klumperman J (2009). Lysosome biogenesis and lysosomal membrane proteins: trafficking meets function. Nat Rev Mol Cell Biol 10:623–35. 19672277 10.1038/nrm2745

[CIT0071] Saftig P (2005). Lysosomes. Boston, MA: Springer.

[CIT0072] Sahay G, Alakhova DY, Kabanov AV (2010). Endocytosis of nanomedicines. J Control Release 145:182–95. 20226220 10.1016/j.jconrel.2010.01.036 PMC2902597

[CIT0073] Sakhtianchi R, Minchin RF, Lee KB, et al. (2013). Exocytosis of nanoparticles from cells: role in cellular retention and toxicity. Adv Colloid Interface Sci 201–202:18–29. 10.1016/j.cis.2013.10.013 24200091

[CIT0074] Salaun C, James DJ, Chamberlain LH (2004). Lipid rafts and the regulation of exocytosis. Traffic 5:255–64. 15030567 10.1111/j.1600-0854.2004.0162.x PMC2394575

[CIT0075] Sato K, Nagai J, Mitsui N, et al. (2009). Effects of endocytosis inhibitors on internalization of human IgG by Caco-2 human intestinal epithelial cells. Life Sci 85:800–7. 19879882 10.1016/j.lfs.2009.10.012

[CIT0076] Schenk M, Mueller C (2008). The mucosal immune system at the gastrointestinal barrier. Best Pract Res Clin Gastroenterol 22:391–409. 18492562 10.1016/j.bpg.2007.11.002

[CIT0077] Schöttler S, Becker G, Winzen S, et al. (2016). Protein adsorption is required for stealth effect of poly (ethylene glycol)-and poly (phosphoester)-coated nanocarriers. Nature Nanotech 11:372–7. 10.1038/nnano.2015.330 26878141

[CIT0078] Serda RE, Mack A, van de Ven AL, et al. (2010). Logic-embedded vectors for intracellular partitioning, endosomal escape, and exocytosis of nanoparticles. Small 6:2691–700. 20957619 10.1002/smll.201000727 PMC2997879

[CIT0079] Settembre C, Fraldi A, Medina DL, Ballabio A (2013). Signals from the lysosome: a control centre for cellular clearance and energy metabolism. Nat Rev Mol Cell Biol 14:283–96. 23609508 10.1038/nrm3565 PMC4387238

[CIT0080] Song Q, Yao L, Huang M, et al. (2012). Mechanisms of transcellular transport of wheat germ agglutinin-functionalized polymeric nanoparticles in Caco-2 cells. Biomaterials 33:6769–82. 22705199 10.1016/j.biomaterials.2012.05.066

[CIT0081] Vllasaliu D, Alexander C, Garnett M, et al. (2012). Fc-mediated transport of nanoparticles across airway epithelial cell layers. J Control Release 158:479–86. 22200577 10.1016/j.jconrel.2011.12.009

[CIT0082] Walczyk D, Bombelli FB, Monopoli MP, et al. (2010). What the cell “sees” in bionanoscience. J Am Chem Soc 132:5761–8. 20356039 10.1021/ja910675v

[CIT0083] Wang JA, Meyer TF, Rudel T (2008). Cytoskeleton and motor proteins are required for the transcytosis of *Neisseria gonorrhoeae* through polarized epithelial cells. Int J Med Microbiol 298:209–21. 17683982 10.1016/j.ijmm.2007.05.004

[CIT0084] Wang L-H, Rothberg KG, Anderson R (1993). Mis-assembly of clathrin lattices on endosomes reveals a regulatory switch for coated pit formation. J Cell Biol 123:1107–17. 8245121 10.1083/jcb.123.5.1107 PMC2119875

[CIT0085] Wang YY, Lai SK, Suk JS, et al. (2008). Addressing the PEG mucoadhesivity paradox to engineer nanoparticles that “slip” through the human mucus barrier. Angew Chem Int Ed 47:9726–9. 10.1002/anie.200803526 PMC2666733 18979480

[CIT0086] Wang Z, Tiruppathi C, Cho J, et al. (2011). Delivery of nanoparticle: complexed drugs across the vascular endothelial barrier via caveolae. IUBMB Life 63:659–67. 21766412 10.1002/iub.485 PMC3142311

[CIT0087] Wang Z, Tiruppathi C, Minshall RD, Malik AB (2009). Size and dynamics of caveolae studied using nanoparticles in living endothelial cells. ACS Nano 3:4110–6. 19919048 10.1021/nn9012274 PMC3643811

[CIT0088] Wiley DT, Webster P, Gale A, Davis ME (2013). Transcytosis and brain uptake of transferrin-containing nanoparticles by tuning avidity to transferrin receptor. Proc Natl Acad Sci USA 110:8662–7. 23650374 10.1073/pnas.1307152110 PMC3666717

[CIT0089] World Health Organization. (2016). Global report on diabetes. Geneva, Switzerland: World Health Organization.

[CIT0090] Yanes RE, Tarn D, Hwang AA, et al. (2013). Involvement of lysosomal exocytosis in the excretion of mesoporous silica nanoparticles and enhancement of the drug delivery effect by exocytosis inhibition. Small 9:697–704. 23152124 10.1002/smll.201201811 PMC3767416

[CIT0091] Ye D, Raghnaill MN, Bramini M, et al. (2013). Nanoparticle accumulation and transcytosis in brain endothelial cell layers. Nanoscale 5:11153–65. 24077327 10.1039/c3nr02905k

[CIT0092] Ye D, Dawson KA, Lynch I (2015). A TEM protocol for quality assurance of *in vitro* cellular barrier models and its application to the assessment of nanoparticle transport mechanisms across barriers. Analyst 140:83–97. 25303735 10.1039/c4an01276c

[CIT0093] Zhang Z, Huang J, Jiang S, et al. (2013). Porous starch based self-assembled nano-delivery system improves the oral absorption of lipophilic drug. Int J Pharm 444:162–8. 23340325 10.1016/j.ijpharm.2013.01.021

[CIT0094] Zhang Z, Huang Y, Gao F, et al. (2011). Daidzein–phospholipid complex loaded lipid nanocarriers improved oral absorption: *in vitro* characteristics and *in vivo* behavior in rats. Nanoscale 3:1780–7. 21350765 10.1039/c0nr00879f

[CIT0095] Zubareva AA, Shcherbinina TS, Varlamov VP, Svirshchevskaya EV (2015). Intracellular sorting of differently charged chitosan derivatives and chitosan-based nanoparticles. Nanoscale 7:7942–52. 25866253 10.1039/c5nr00327j

